# Survival outcomes after surgical resection of huge HCC (≥ 10 cm) with or without neoadjuvant hepatic arterial infusion chemotherapy

**DOI:** 10.1186/s12957-025-03876-1

**Published:** 2025-06-23

**Authors:** Chia-Ling Chiang, Yu-Chia Chen, Huei-Lung Liang, Cheng-Chung Tsai, I-Shu Chen, Gu-Sheng Yan, Wei-Lun Tsai

**Affiliations:** 1 Department of Radiology, Kaohsiung Veterans General Hospital, 386 Dazhong 1st Road, Zuoying, Kaohsiung, 813 Taiwan, ROC; 2 Interventional Center, Antai Tian-Sheng Memorial Hospital, Kaohsiung, Taiwan; 3 Department of Surgery, Kaohsiung Veterans General Hospital, Kaohsiung, Taiwan, ROC; 4 Division of Gastroenterology and Hepatology, Department of Medicine, Kaohsiung Veterans General Hospital, Kaohsiung, Taiwan, ROC; 5 Department of Radiology, National Yang Ming Chiao Tung University, Hsinchu, Taiwan; 6 Shu-Zen Junior College of Medicine and Management, Kaohsiung, Taiwan, ROC

**Keywords:** Hepatocellular carcinoma, Neoadjuvant hepatic arterial infusion chemotherapy, Surgical resection, Disease-free survival, Tumor recurrence

## Abstract

**Purpose:**

To evaluate the survival outcomes of huge HCC (tumor size ≥ 10 cm) after surgical resection (SR) with or without neoadjuvant hepatic arterial infusion chemotherapy (HAIC).

**Patients and methods:**

119 huge HCC patients underwent SR in our Hospital (2010–2020). A new HAIC regimen (cisplatin, leucovorin, mitomycin-C and 5-FU infusion for 5 days plus 10 ml lipiodol microvascular embolization) was adopted as the neoadjuvant therapy in 25 patients. Treatment responses were evaluated based on mRECIST criteria. The objective response rate (ORR), disease free survival (DFS), recurrence survival (RS) and overall survival (OS) were compared between the SR-only and neoadjuvant HAIC groups.

**Results:**

Of the 119 patients, 65 patients were Vp2, 9 patients were Vp3 and 4 patients were Vp4. In the subgroup analysis, neoadjuvant HAIC group revealed significantly more severe clinical status. Of the neoadjuvant HAIC patients, ORR was 66.7%. Postoperative tumor recurrence was noted in 75% and 58.3% of the SR and neoadjuvant groups, of them 56.5% and 20.8% developed in ≤ 12 months. The median DFS, RS and OS in each group were 10 vs. 41 months (*p* = 0.016), 36 vs. 91 months and 46 vs. 96 months, respectively. Subgroup analysis revealed no significant survival difference of the RS in both patient groups with tumor recurrence ≤ 12 months (17 vs. 14 months) or > 12 months/without recurrence (not reached vs. 113 months).

**Conclusion:**

Our new regimen HAIC acted as an effective neoadjuvant therapy in reducing early recurrence rate and prolonged DFS of huge HCC after surgical resection.

## Introduction

Hepatocellular carcinoma (“HCC”) is the sixth most common cancer and the third leading cause of cancer mortality worldwide [1]. Surgical resection (“SR”), liver transplantation (“LT”), and local ablation are recommended as the definitive therapy for early stage HCC, with a reported 5-year survival rate of 60-80% [2]. Although tumor size per se is not currently considered an important predictor of survival– reflected by the omission of size as a prognostic variable in recent editions of the Barcelona Clinic Liver Cancer (“BCLC”) and the American Joint Committee on Cancer’s “TNM” staging systems (“AJCC TNM”) staging [3–5]– the overall survival (OS) of huge HCCs (defined as ≥ 10 cm) after SR has been reported as poorer than those of smaller than 10 cm, with a 5-year OS of 40.0 or 35.6% vs. 65.9 or 62.7%, respectively [6, 7]. The poorer prognoses of huge HCCs are largely attributed to the fact that larger tumors are more frequently associated with other adverse prognostic factors, such as vascular invasion, multifocality, and higher grades [4].

Neoadjuvant therapy is a multidisciplinary treatment for malignancies with the aim of preventing tumor progression or even downstaging solid tumors [8]. With neoadjuvant therapy, the success rate in downstaging HCC within the Milan criteria can be more than 60% in selected patients [9]. Some clinical studies have confirmed that patients who underwent LT or SR after successful downstaging/conversion treatment achieved prognoses similar to that of patients without such treatment [9–13].

Hepatic artery infusion chemotherapy (HAIC) has been proven to be an effective treatment for advanced HCC [14–17]. In Liu et al.’s meta-analysis, they found better OS and progression-free survival (PFS) for the HAIC group compared to the sorafenib group in HCC with portal vein tumor thrombosis (PVTT) [15]. Our own early study of SR for huge HCC patients before 2000 showed that the median OS was 17.0 months [18]. We later developed a new regimen of HAIC (continuous chemoinfusion plus lipiodol microvascular embolization) in treating advanced HCC, which can also be used as a neoadjuvant for conversion treatment or post-SR adjuvant therapy. In this study, we evaluated the survival outcome of huge HCC treated by SR, with or without our new neoadjuvant HAIC procedure.

## Methods

The data of all patients presenting at Kaohsiung Veterans General Hospital with newly diagnosed huge HCC and who underwent SR with or without neoadjuvant HAIC therapy between 2010 and 2020 were retrospectively collected and analyzed. Collated data included patient characteristics such as age, gender, presence of hepatitis B or C virus (“HBV” or “HCV”) infection, and relevant radiological and biochemical investigations. Patients with major portal vein invasion (“MPVI”) or extrahepatic spread (“EHS”) were not absolutely excluded for SR in our hospital as intrahepatic tumor progression was the main cause of advanced HCC-related deaths. If intrahepatic HCC could be controlled, it could significantly increase overall survival. The severity of PVTT was categorized according to the Japanese “VP” Staging Classification System [19], where Vp4 was defined as the presence of a tumor thrombus in the main trunk or a portal vein branch contralateral to the primarily involved lobe; Vp3 and Vp2 as tumor invasion of the first-order and second-order branches respectively; and Vp1 and Vp0 as no visible vascular invasion on images. Patients who expired within 30 days after SR were recorded as peri-operative mortality and were excluded from further statistical analysis for disease free survival (DFS), recurrence survival (RS) and overall survival (OS).

This study was conducted in accordance with the Declaration of Helsinki. Written informed consent was obtained from each patient after detailed explanation about the therapy. This retrospective study was approved by the ethics review committee of Kaohsiung Veterans General Hospital with patient informed consent waived for this retrospective study (VGHKS22-CT7-03). These eligible subjects were followed until July 2024.

### Surgical resection (SR)

SR was offered to patients with a resectable tumor, provided that functional reserve was sufficient. Tumors invading ipsilateral hepatic or portal veins, and tumors with satellite nodules in the same liver lobe were also considered for resection. Disseminated metastasis was excluded first while the peritoneal cavity was entered. Then the degree of PV/HV invasion were checked via intraoperative ultrasound. Anatomical resections were usually applied for this kind of huge tumors. Ischemic demarcation was confirmed after appropriate PV/HA clamping. Parenchymal transection was done using Cavitron Ultrasonic Surgical Aspirator (CUSA; Valleylab Inc., Boulder, Colorado), Harmonic scalpel (Ethicon Endo-Surgery, Johnson & Johnson), Sonicision (Covidien), Kelly clamp crushing or other energy devices.

### Hepatic arterial infusion chemotherapy (HAIC)

HAIC was performed via puncture of the left subclavian (axillary) artery under ultrasonographic guidance. Our “new” HAIC regimen consisted of a daily pump-infusion of 10mg/m^2^ cisplatin, 2mg/m^2^ mitomycin-C and 15 mg/m^2^ Leucovorin, each administered for 20 to 30 min, plus a slow infusion (22 h) of 100mg/m^2^ 5-fluorouracil (5-FU) for 5 days. After completion of the 5-day’s chemo-infusion, we injected 10 ml Lipiodol (Guerbet, France) into the hepatic artery. The angio-catheter was then removed on the 5th treatment day. This neoadjuvant HAIC was performed for at least two courses. The timing of SR for the residual HCC after neoadjuvant HAIC was up to the surgeons’ discretion.

### Response assessment and survival

As HAIC is a locoregional therapy, intrahepatic tumor response was used to judge HAIC treatment efficacy, rather than whole disease response based on dynamic CT or MRI liver images after every 2 courses of HAIC. Treatment efficacy was classified into complete response (CR), partial response (PR), stable disease (SD), and progressive disease (PD) according to the Modified Response Evaluation Criteria in Solid Tumors (mRECIST) guidelines [20]. The objective response rate was defined as CR plus PR.

Disease free survival (DFS) was defined as the time interval between the operation date in the SR group or the HAIC date in the neoadjuvant HAIC group and the tumor recurrence date. Recurrence survival (RS) was defined as the time interval between the recurrence date and the last follow-up date. Overall survival (OS) was defined as the time interval between the operation date or HAIC date and the last follow-up date in each group.

### Statistical analysis

Baseline patient characteristics were presented using descriptive statistics, presented as mean ± standard deviation. Nominal data were compared using Student’s t-test. DFS, RS and OS were computed using Kaplan–Meier analysis, and differences in survival curves were compared using log-rank testing. All statistical analyses were performed with SPSS version 26.0. A *p*-value of less than 0.05 was considered statistically significant.

## Results

### Patient demographics

A total of 119 huge HCC (≥ 10 cm) patients (104 male and 15 female with the mean age of 61.4 ± 13.8 years) received SR in our hospital during 2010–2020. Of these patients, 25 had undergone neoadjuvant HAIC therapy before SR. HBV infection was the most common cause (60.5%), followed by non-B, non-C viral infection (23.5%), and chronic HCV infection (16%). 49 (41.2%) patients had alpha-fetal protein ≥ 400 ng/mL. The mean maximal tumor size was 13.5 ± 2.8 cm. 10 (8.4%) patients had more than 3 tumors. 100 (84.0%) patients had unilobar involvement (64.7% in the right lobe and 19.3% in the left lobe), while bilateral lobe involvement was seen in 19 (16%) patients. 44 (37%) of the 119 patients had no imaging evidence of portal vascular invasion. 62 (52.1%) patients were classified as Vp2; and13 (10.9%) patients had MPVI, with 9 such patients classified as Vp3 and 4 classified as Vp4. 4 patients had concurrent hepatic vein/IVC invasion. Extrahepatic spread (lymph node, lung, and bone) was found at the initial diagnosis in 10 (8.4%) patients. Table 1 summarizes the demographic data of the 119 huge HCC and the subgroups of the SR and neoadjuvant HAIC group. In the subgroup analysis, neoadjuvant HAIC group revealed significantly more “severe” clinical statuses in tumor number, bilateral lobe involvement, portal vein invasion, extrahepatic spread and combined hepatic vein invasion.

**Table 1:** Basic demographic data of the huge HCC patients

Variable	overall	surgical resection	neoadjuvant HAIC	*p* value
Patients	119	94	25	
Age (years old, mean ± SD)	61.4 ± 13.8	62.3 ± 14.4	57.9 ± 11.1	0.112
Male/Female, n (%)	104/15 (87.4/12.6)	81/13 (86.2/13.8)	23/2 (92/8)	0.74
Etiology, n (%)				
HBV/HCV/non-BC	72/19/28 (60.5/16/23.5)	54/16/24 (57.4/17/25.5)	18/3/4 (72/12/16)	0.41
AFP ≧ 400 ng/mL, n (%)	49 (41.2)	36 (38.3)	13 (52)	0.22
Maximal tumor size (cm)	13.5 ± 2.8	13.6 ± 2.6	11.1 ± 3.2	0.47
Tumor number (> 3)	10 (8.4)	4 (4.3)	6 (24)	0.006*
Tumor involvement, n (%)				0.001*
Unilobar(R/L)	77/23 (64.7/19.3)	63/22 (67/23.4)	14/1 (56/4)	
Bilobar	19 (16)	9 (9.6)	10 (40)	
Portal vein invasion				0.004*
Vp0/1	44 (37)	39 (41.5)	5 (20)	
Vp2	62 (52.1)	49 (52.1)	13 (52)	
Vp3/4	13 (10.9)	6 (6.4)	7 (28)	
Extrahepatic metastasis	10 (8.4)	5(5.3)	5 (20)	0.033*
Combined HV invasion	4 (3.4)	1 (1.1)	3 (12)	0.029*

### Clinical outcome and long-term survival of huge HCC after SR

**Fig. 1:**
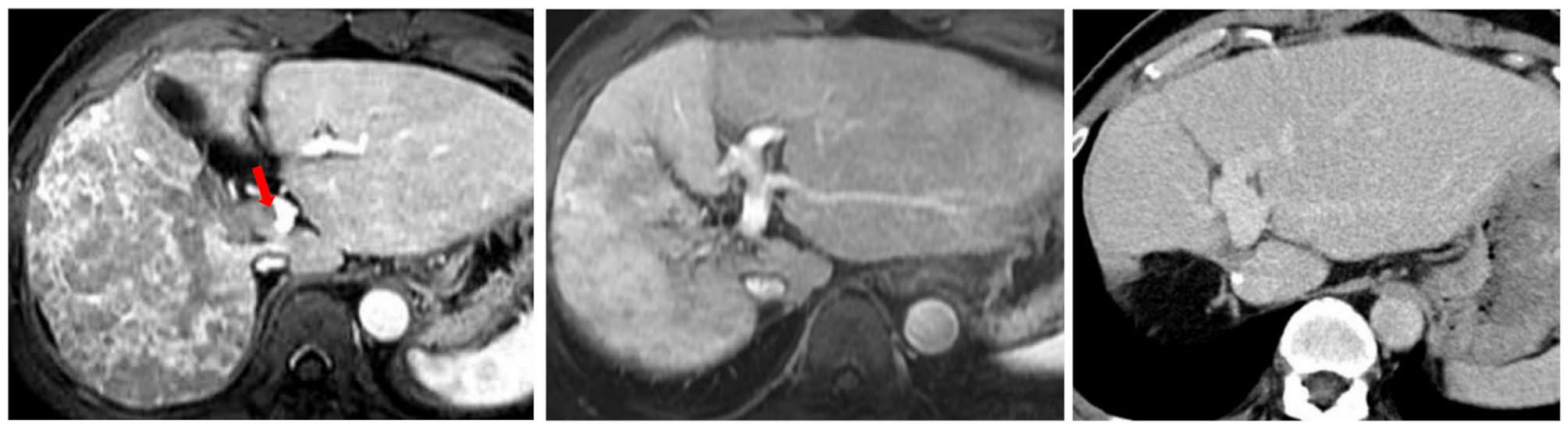
A 56 year-old male patient with a confluent 14 cm HCC in the right lobe liver. **A**: Red arrow indicates tumor invasion into the main portal vein (Vp4). **B**: After 3 courses of HAIC neoadjuvant therapy with PR, this patient underwent right hepatectomy. **C**: 5-year follow-up CT image showed no evidence of intrahepatic recurrence

The perioperative surgical mortality rate was 2.1% (2/94) in the SR-only group and 4% (1/25) in the neoadjuvant group. After excluding these 3, the remaining 116 patients were followed for a mean period of 56.9 ± 43.9 months. Of the 24 neoadjuvant HAIC patients, CR was achieved in 4 (16.7%) patients, PR in 12 (50%) patients (Fig. 1), SD in 7 (29.2%) patients and PD in 1 (4.2%) patient, respectively.

**Fig. 2:**
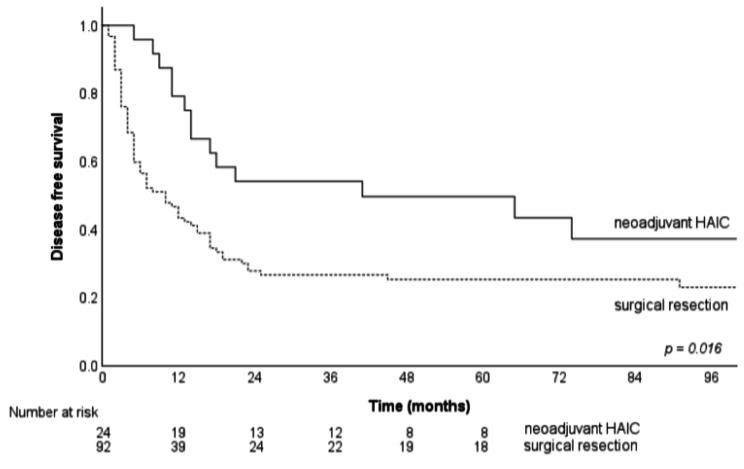
The DRS of the SR alone and neoadjuvant HAIC group patients

69 of the 92 (75%) SR-only group patients developed tumor recurrence during postoperative follow-up; of these patients, 52 (56.5%) had tumor recurrence ≤ 12months. 14 of the 24 (58.3%) neoadjuvant HAIC patients developed tumor recurrence during the postoperative follow-up period, with tumor recurrence ≤ 12months in 5 patients (20.8%). The median and 1-, 3-, and 5-year DFS in each group were 10 vs. 41 months, 44.5% vs. 75%, 25.3% vs. 49.7%, and 23% vs. 43.4%, respectively (*p* = 0.016) (Fig. 2).

**Fig. 3:**
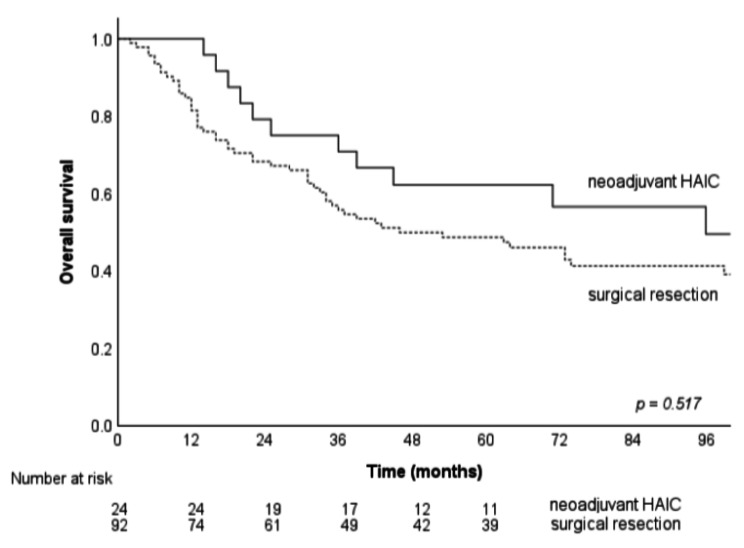
The OS of the SR-only and neoadjuvant HAIC group patients

The median and 1-, 3-, and 5-year OS rates of the SR-only and neoadjuvant HAIC groups were 46 vs. 96 months, 81.5% vs. 95.8%, 55.8% vs. 70.8% and 47.7% vs. 56.6% (*p* = 0.517) (Fig. 3).

**Fig. 4:**
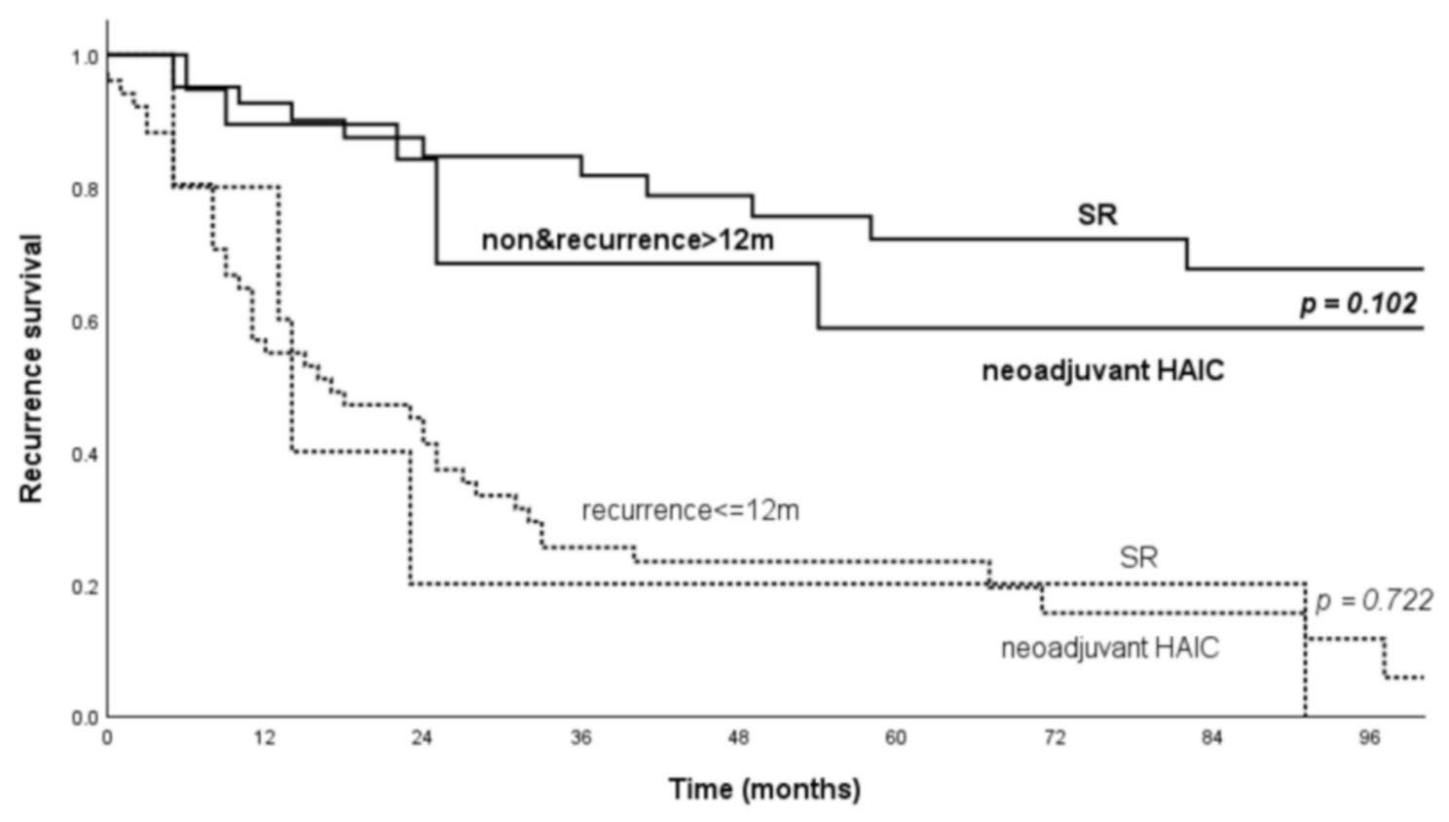
Subgroup analysis of RS (≤ or > 12 months) in the SR-only and neoadjuvant HAIC group patients

The median and 1-, 3-, and 5-year RS of the SR-only and neoadjuvant HAIC groups was 36 vs. 91 months, 71.7% vs. 83.3%, 49.4% vs. 51% and 42.4% vs. 42.5%, respectively (*p* = 0.69). Subgroup analysis of the RS revealed that the median RS in the SR and neoadjuvant group were 17 vs. 14 months (*p* = 0.722) in patients with tumor recurrence ≤ 12 months and “not reached” vs. 113 months (*p* = 0.102) in patients with tumor recurrence > 12 months or without tumor recurrence (Fig. 4).

**Fig. 5:**
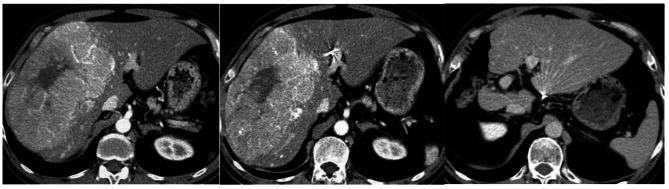
A 54 year-old male advanced HCC patient. **A**: CT image showed a huge HCC (16 cm) in the right lobe liver with right segmental portal vein invasion (not shown). **B**: After 2 courses of HAIC neoadjuvant therapy with SD, this patient underwent right hepatectomy. **C**: 10-year follow-up CT image showed no evidence of intrahepatic recurrence

**Fig. 6:**
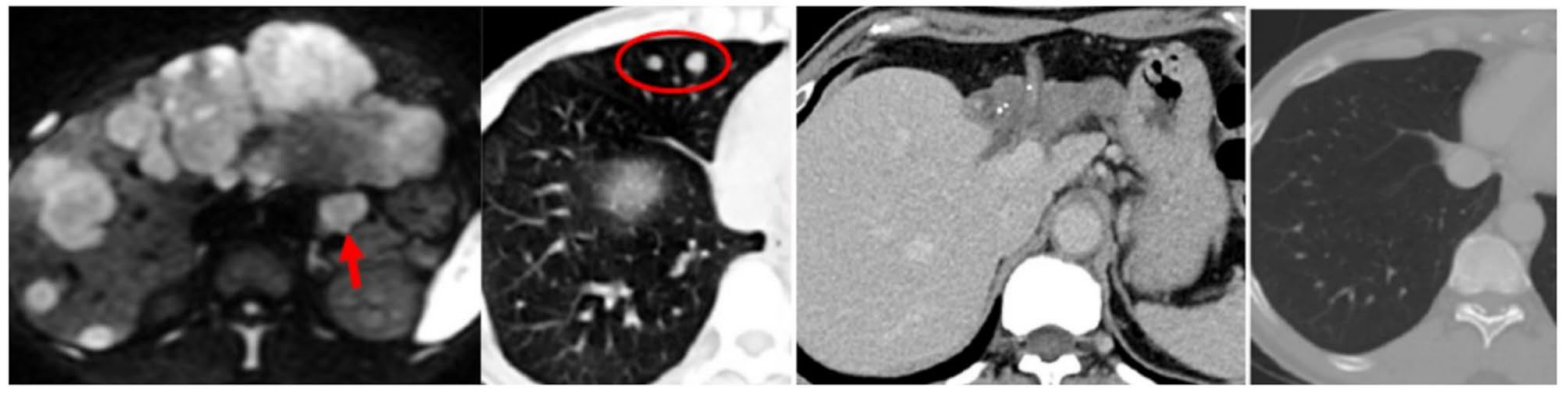
A 50 year-old male advanced HCC patient with extrahepatic spread. **A**: MRI of the liver (diffusion-weighted image) showed diffuse multiple HCC in the bilateral lobe of liver with a metastatic lymph node (red arrow) in the gastrohepatic ligament. **B**: CT image of the chest showed several metastatic nodules in the right lung field (red circle). **C**: CT image of the liver showed complete response of the left lobe lesions with non-visible metastatic lymph node after HAIC neoadjuvant therapy. **D**: CT image of the chest showed disappearance of the right lung nodules after HAIC treatment. This patient underwent left hepatectomy and resection of the residual viable lesion in the right lobe liver with disease free survival for more than 70 months till the end of the study

For the patients with MPVI, the median OS of the 6 SR-only patients was 11 months while “not reached” in the 7 neoadjuvant patients by the end of this study (July 2024). For the SD patients, 3 (42.9%) of the 7 had no tumor recurrence after neoadjuvant HAIC therapy (Fig. 5), with a median OS of 45 months. At the end of this study, 27 (29.3%) patients of the SR-only group and 9 (37.5%) patients of the neoadjuvant HAIC group were still alive. A HCC patient with extrahepatic spread (lymph node and lung) after neoadjuvant HAIC was also demonstrated in Fig. 6.

## Discussion

For patients with huge (≥ 10 cm) HCC, SR and LT offer the best survival outcomes and are the only potentially curative modalities [4]. Nonetheless, the HCC tumor recurrence rate remains high, and long-term survival remains modest after SR [7, 21–24]. The median OS and 5-year survival of huge HCC after SR are reported in the literature as between 17 and 60 months and 16.7–58% respectively [4, 6, 7, 18, 21, 22, 24–29], with the 12-month recurrence rate between 51% and 71.7% [18, 21, 24, 25, 29]. Our data before 2000 revealed poorer clinical outcomes of huge HCC after SR with a 12-month recurrence rate of 71.7%, median OS of 17 months, and 5-year OS of 24.5% [18]. In the present study between 2010 and 2020, although the 12-month recurrence rate after SR-only was still high (56.5%), both the median OS and 5-year survival rate improved (46 months and 47.7%). This may be attributed to the development of more effective locoregional and/or systemic therapy for the treatment of tumor recurrence since the turn of the century.

The present study also showed that if tumor recurrence occurred within 12 months after SR (which may be regarded as intrahepatic spread during SR), the clinical outcome was poor with median OS of 33.8 months, versus 137.4 months in patients with tumor recurrence *after* 12 months post-SR (usually regarded as de-novo tumor growth) or in patients without tumor recurrence. Thereafter, it is crucial to postpone or prevent early tumor recurrence after SR for huge HCC.

The purposes of neoadjuvant therapy for HCC are (1) to prevent patients from dropping out for LT (bridging) due to tumor progression during the waiting period [30], (2) to shrink or reduce tumors outside the Milan criteria to meet the indications for LT (downstaging) [31], (3) to increase the SR rate of HCC to convert unresectable HCCs into resectable tumors (conversion) [32], and (4) to prevent new recurrence after radical treatment [33]. Neoadjuvant therapies for HCC include transcatheter embolization (TACE), radiotherapy (RT), ablation therapy, chemotherapy, targeted therapy and immunotherapy and/or combination therapy [34].

For resectable HCC (BCLC A/B), Li et al. reported a retrospective multicenter study of neoadjuvant TACE for huge HCC (≥ 10 cm) without macrovascular invasion [35]. The median OS was respectively 32.8 vs. 22.3 months (*p* = 0.035) between patients with and without preoperative TACE. A systematic review [36] focusing on neoadjuvant TACE for resectable HCC concluded that only one study with solitary large HCC (mean tumor size 9.5 cm vs. 9.9 cm) demonstrated a longer 5-year survival in the neoadjuvant group compared to the SR group (46.2% vs. 31.7%, *p* = 0.043) [37]; the other studies that were surveyed either failed to demonstrate significant improved clinical outcomes or reported poorer outcomes.

The present neoadjuvant HAIC study with a new regimen revealed a promising clinical outcome with a much lower 12-month recurrence rate of 20.8%, compared with the 56.5% of SR-only group. Although the difference between the survival curves between SR-only and neoadjuvant HAIC group patients was not significant (5-year: 47.7% vs. 56.6% respectively, *p* = 0.517) the differences between median DFS (10 vs. 41 months respectively, *p* = 0.016) was significant and the OS was 46 vs. 96 months respectively. This can be explained by the fact that the RS in these two groups showed no difference between patients with tumor recurrence ≤ 12 months and patients without recurrence or recurrence after 12 months. The demonstrated benefit of neoadjuvant HAIC in this study is that the early (≤ 12 months) recurrence rate was markedly reduced (58.3% with SR-only vs. 20.8% with neoadjuvant HAIC), and once early recurrence occurred, poor RS ensued. Based on the above results, it can be inferred that pre-operative HAIC is an effective neoadjuvant therapy.

For vascular-invaded HCC, Chong et al. [38] and Hamaoka et al. [13] reported 116 Vp2/3 and 52 Vp3/4 patients who underwent neoadjuvant RT plus HAIC with successful conversion to SR in 22.4% and 17.3% of the patients, respectively. In both studies, the OS differed significantly between the neoadjuvant CCRT group and the resection-first group; in Chong’s series, the median OS was 62 months versus 15 months (*p* = 0.006), and in Hamaoka’s study, the 3-year OS rate was 71% versus 18% (*p* = 0.009). Salem et al. [39] reported 257 BCLC C Child-Pugh (CP) A patients underwent transarterial radioembolization (TARE) with successful conversion/downstaging in 16%, and the “intent-to treat survival” of the whole CP A cohort was 16.6 months. Tabone et al. reported the clinical outcomes of 24 HCC patients with initially unresectable portal vein invasion receiving neoadjuvant TARE [40], with a successful conversion rate of 20% with a median OS of 70 months. In our present study, 58.5% of the SR group and 80% of the neoadjuvant HAIC group had macrovascular portal vein invasion. Neoadjuvant HAIC seemed effective in reducing tumor spread during SR in HCC patients with vascular invasion. The objective response rate of our 24 neoadjuvant HAIC patients was 66.7% (CR: 16.7% plus PR: 50%), which could be regarded as a successful conversion rate. Notably, the 7 (29.2%) SD patients after neoadjuvant HAIC presented clinical outcomes not inferior to those of SR-only group, with recurrence-free rates of 42.9% vs. 25% and median OS of 45 vs. 46 months, respectively. This implies that the concern of delaying patients for surgical resection in the non-responded neoadjuvant HAIC group is not problematic.

### Limitation

There were some limitations in this study. First, this was a retrospective review with single institute patient cohort. Second, the patient number in the neoadjuvant HAIC group was small, requiring a large series confirmation. Third, the OS was calculated from the HAIC treatment date in the neoadjuvant group instead of from the operative date as in the SR group. Fourth, we didn’t perform propensity-score matching analysis between these two groups; however, in the present study, the severities (including tumor number, bilateral lobe involvement, portal vein invasion, extrahepatic spread and combined hepatic vein invasion) were poorer in the neoadjuvant group. Finally, the present study did not compare the clinical outcomes of neoadjuvant conversion for SR with downstaging for LT.

## Conclusion

Neoadjuvant HAIC is effective in reducing the early recurrence rate of huge HCC after surgical resection, especially of in macrovascular invasion HCC. It seems that both successfully converted patients and stable-disease patients may benefit from neoadjuvant HAIC therapy.

## Data Availability

No datasets were generated or analysed during the current study.

## References

[1] Llovet JM, Pinyol R, Yarchoan M, Singal AG, Marron TU, Schwartz M, et al. Adjuvant and neoadjuvant immunotherapies in hepatocellular carcinoma. Nat Reviews Clin Oncol. 2024;21(4):294–311. 10.1038/s41571-024-00868-0 10.1038/s41571-024-00868-0 PMC11984461 38424197

[2] European Association for the Study of the Liver. EASL clinical practice guidelines: management of hepatocellular carcinoma. J Hepatol. 2018;69:182–236. 10.1016/j.jhep.2018.03.019]. [PMID: 29628281. 29628281 10.1016/j.jhep.2018.03.019

[3] American Joint Committee on Cancer. In. In: Edge SB, Byrd DR, Compton CC, et al. editors. American joint committee on Cancer staging manual, 7th. New York: Springer; 2010. p. 175.

[4] Goh BK, Kam JH, Lee SY, et al. Significance of neutrophil-to-lymphocyte ratio, platelet-to-lymphocyte ratio and prognostic nutrition index as preoperative predictors of early mortality after liver resection for huge (≥ 10 cm) hepatocellular carcinoma. J SurgOncol. 2016;113(6):621–7. 10.1002/jso.24197 10.1002/jso.24197 26861568

[5] Reig M, Forner A, Rimola J et al. BCLC strategy for prognosis prediction and treatment recommendation: the 2022 update. J Hepatol. 2022;76(3):681–93. 10.1016/j.jhep.2021.11.018 10.1016/j.jhep.2021.11.018 PMC8866082 34801630

[6] Choi GH, Han DH, Kim DH, et al. Outcome after curative resection for a huge (> or = 10 cm) hepatocellular carcinoma and prognostic significance of gross tumor classification. Am J Surg. 2009;198(5):693–701. 10.1016/j.amjsurg.2008.09.019 19268907 10.1016/j.amjsurg.2008.09.019

[7] Chen JH, Wei CK, Lee CH, Chang CM, Hsu TW, Yin WY. The safety and adequacy of resection on hepatocellular carcinoma larger than 10 cm: a retrospective study over 10 years. Ann Med Surg (Lond). 2015;4(2):193–9. 10.1016/j.amsu.2015.05.003. Published 2015 May 14. 26052436 10.1016/j.amsu.2015.05.003 PMC4454785

[8] Fujiki M, Aucejo F, Choi M, Kim R. Neo-adjuvant therapy for hepatocellular carcinoma before liver transplantation: where do we stand? World J Gastroenterol. 2014;20:5308–19. 10.3748/wjg.v20.i18.5308]. [PMID: 24833861. 24833861 10.3748/wjg.v20.i18.5308 PMC4017046

[9] Lei J, Wang W, Yan L. Downstaging advanced hepatocellular carcinoma to the Milan criteria may provide a comparable outcome to conventional Milan criteria. J Gastrointest Surg. 2013;17:1440–6. [PMID: 23719776 DOI: 10.1007/s11605-013-2229-y]. 23719776 10.1007/s11605-013-2229-y

[10] Lau WY, Lai EC. Salvage surgery following downstaging of unresectable hepatocellular carcinoma strategy to increase resectability. Ann Surg Oncol. 2007;14:3301–9. 10.1245/s10434-007-9549-7]. [PMID: 17891443 DOI:. 17891443 10.1245/s10434-007-9549-7

[11] Pompili M, Francica G, Ponziani FR, Iezzi R, Avolio AW. Bridging and downstaging treatments for hepatocellular carcinoma in patients on the waiting list for liver transplantation. World J Gastroenterol. 2013;19:7515–30. 10.3748/wjg.v19.i43.7515]. [PMID: 24282343. 24282343 10.3748/wjg.v19.i43.7515 PMC3837250

[12] Finkenstedt A, Vikoler A, Portenkirchner M, Mülleder K, Maglione M, Margreiter C, et al. Excellent post-transplant survival in patients with intermediate stage hepatocellular carcinoma responding to neoadjuvant therapy. Liver Int. 2016;36:688–95. 10.1111/liv.12966]. [PMID: 26386273. 26386273 10.1111/liv.12966

[13] Hamaoka M, Kobayashi T, Kuroda S, Iwako H, Okimoto S, Kimura T, et al. Hepatectomy after down-staging of hepatocellular carcinoma with portal vein tumor thrombus using chemoradiotherapy: a retrospective cohort study. Int J Surg. 2017;44:223–8. 10.1016/j.ijsu.2017.06.082 28676383 10.1016/j.ijsu.2017.06.082

[14] Zhuang BW, Li W, Xie XH, Hu HT, Lu MD, Xie XY. Sorafenib versus hepatic arterial infusion chemotherapy for advanced hepatocellular carcinoma: a systematic review and meta-analysis. Jpn J Clin Oncol. 2019;49(9):845–55. 31063184 10.1093/jjco/hyz069

[15] Liu M, Shi J, Mou T, Wang Y, Wu Z. Systematic review of hepatic arterial infusion chemotherapy versus sorafenib in patients with hepatocellular carcinoma with portal vein tumor thrombosis. J Gastroenterol Hepatol. 2020;35(8):1277–87. 32052876 10.1111/jgh.15010

[16] Zheng K, Zhu X, Fu S, Cao G, Li WQ, Xu L, et al. Sorafenib plus hepatic arterial infusion chemotherapy versus sorafenib for hepatocellular carcinoma with major portal vein tumor thrombosis. Randomized Trial Radiol. 2022;303(2):455–64. 10.1148/radiol.211545 35103539

[17] He M, Li Q, Zou R, Shen JX, Fang WQ, Tan GS, et al. Sorafenib plus hepatic arterial infusion of oxaliplatin, fluorouracil, and leucovorin vs sorafenib alone for hepatocellular carcinoma with portal vein invasion: a randomized clinical trial. JAMA Oncol. 2019;5(7):953–60. 31070690 10.1001/jamaoncol.2019.0250 PMC6512278

[18] Mok KT, Wang BW, Lo GH, et al. Multimodality management of hepatocellular carcinoma larger than 10 cm. J Am Coll Surg. 2003;197(5):730–8. 10.1016/j.jamcollsurg.2003.07.013 14585406 10.1016/j.jamcollsurg.2003.07.013

[19] Shi J, Lai ECH, Li N, Guo WX, Xue J, Lau WY, et al. A new classification for hepatocellular carcinoma with portal vein tumor thrombus. J Hepatobiliary Pancreat Sci. 2011;18(1):74–80. 20686792 10.1007/s00534-010-0314-0

[20] Lencioni R, Eric CH, Lai N, Li, Guo WX, Xue J, Lau WY, et al. Objective response by mRECIST as a predictor and potential surrogate end-point of overall survival in advanced. HCC J Hepatol. 2017;66(6):1166–72. 28131794 10.1016/j.jhep.2017.01.012

[21] Shrager B, Jibara GA, Tabrizian P, Schwartz ME, Labow DM, Hiotis S. Resection of large hepatocellular carcinoma (≥ 10 cm): a unique Western perspective. J SurgOncol. 2013;107(2):111–7. 10.1002/jso.23246 10.1002/jso.23246 22903563

[22] Lim C, Compagnon P, Sebagh M, et al. Hepatectomy for hepatocellular carcinoma larger than 10 cm: preoperative risk stratification to prevent futile surgery. HPB (Oxford). 2015;17(7):611–23. 10.1111/hpb.12416 25980326 10.1111/hpb.12416 PMC4474509

[23] Chan YC, Kabiling CS, Pillai VG, et al. Survival outcome between hepatic resection and transarterial embolization for hepatocellular carcinoma more than 10 cm: a propensity score model. World J Surg. 2015;39:1510–8. 25665673 10.1007/s00268-015-2975-y

[24] Hwang S, Lee YJ, Kim KH, et al. Long-term outcome after resection of huge hepatocellular carcinoma ≥ 10 cm: single-institution experience with 471 patients. World J Surg. 2015;39(10):2519–28. 10.1007/s00268-015-3129-y 26126423 10.1007/s00268-015-3129-y

[25] Yeh CN, Lee WC, Chen MF. Hepatic resection and prognosis for patients with hepatocellular carcinoma larger than 10 cm: two decades of experience at Chang Gung memorial hospital. Ann SurgOncol. 2003;10(9):1070–6. 10.1245/aso.2003.03.072 10.1245/aso.2003.03.072 14597446

[26] Pandey D, Lee KH, Wai CT, et al. Long term outcome and prognostic factors for large hepatocellular carcinoma (10 cm or more) after surgical resection. Ann SurgOncol. 2007;14:2817–23. 10.1245/s10434-007-9518-1 10.1245/s10434-007-9518-1 17690940

[27] Min YW, Lee JH, Gwak GY, et al. Long-term survival after surgical resection for huge hepatocellular carcinoma: comparison with transarterial chemoembolization after propensity score matching. J GastroenterolHepatol. 2014;29(5):1043–8. 10.1111/jgh.12504 10.1111/jgh.12504 24863186

[28] Lim C, Mise Y, Sakamoto Y, et al. Above 5 cm, size does not matter anymore in patients with hepatocellular carcinoma. World J Surg. 2014;38(11):2910–8. 10.1007/s00268-014-2704-y 25099682 10.1007/s00268-014-2704-y

[29] Lee CW, Yu MC, Wang CC, Lee WC, Tsai HI, Kuan FC, Chen CW, Hsieh YC, Chen HY. Liver resection for hepatocellular carcinoma larger than 10 cm: a multi-institution long-term observational study. World J Gastrointest Surg. 2021;13(5):476–92. 10.4240/wjgs.v13.i5.476. PMID: 34122737; PMCID: PMC8167847. 34122737 10.4240/wjgs.v13.i5.476 PMC8167847

[30] Kollmann D, Selzner N, Selzner M. Bridging to liver transplantation in HCC patients. Langenbecks Arch Surg. 2017;402:863–71. [PMID: 28755240 DOI: 10.1007/s00423-017-1609-2]. 28755240 10.1007/s00423-017-1609-2

[31] Mazzaferro V, Citterio D, Bhoori S, Bongini M, Miceli R, De Carlis L, et al. Liver transplantation in hepatocellular carcinoma after tumour downstaging: a randomised, controlled, phase 2b/3 trial. Lancet Oncol. 2020;21:947–56. [PMID: 32615109 DOI: 10.1016/S1470-2045(20)30224-2]. 32615109 10.1016/S1470-2045(20)30224-2

[32] Zhang ZF, Luo YJ, Lu Q, Dai SX, Sha WH. Conversion therapy and suitable timing for subsequent salvage surgery for initially unresectable hepatocellular carcinoma: what is new? World J Clin Cases. 2018;6:259–73. 10.12998/wjcc.v6.i9.259]. [PMID: 30211206. 30211206 10.12998/wjcc.v6.i9.259 PMC6134280

[33] Xu M, Doyle MM, Banan B, Vachharajani N, Wang X, Saad N, Fowler K, Brunt EM, Lin Y, Chapman WC. Neoadjuvant locoregional therapy and recurrent hepatocellular carcinoma after liver transplantation. J Am Coll Surg. 2017;225:28–40. 10.1016/j.jamcollsurg.2017.03.015]. [PMID: 28400300. 28400300 10.1016/j.jamcollsurg.2017.03.015 PMC5777313

[34] Akateh C, Black SM, Conteh L, Miller ED, Noonan A, Elliott E, Pawlik TM, Tsung A, Cloyd JM. Neoadjuvant and adjuvant treatment strategies for hepatocellular carcinoma. World J Gastroenterol. 2019;25:3704–21. 10.3748/wjg.v25.i28.3704]. [PMID: 31391767. 31391767 10.3748/wjg.v25.i28.3704 PMC6676544

[35] Li C, Wang MD, Lu L, et al. Preoperative transcatheter arterial chemoembolization for surgical resection of huge hepatocellular carcinoma (≥ 10 cm): a multicenter propensity matching analysis. Hepatol Int. 2019;13(6):736–47. 10.1007/s12072-019-09981-0 31486964 10.1007/s12072-019-09981-0

[36] Chua TC, Liauw W, Saxena A, Chu F, Glenn D, Chai A, et al. Systematic review of neoadjuvant transarterial chemoembolization for resectable hepatocellular carcinoma. Liver Int. 2010;30:166e74. 19912531 10.1111/j.1478-3231.2009.02166.x

[37] Chen XP, Hu DY, Zhang ZW, Zhang BX, Chen YF, Zhang WG, et al. Role of mesohepatectomy with or without transcatheter arterial chemoembolization for large centrally located hepatocellular carcinoma. Dig Surg. 2007;24:208e13. 17522469 10.1159/000102901

[38] Chong JU, Choi GH, Han DH, et al. Downstaging with localized concurrent chemoradiotherapy can identify optimal surgical candidates in hepatocellular carcinoma with portal vein tumor thrombus. Ann Surg Oncol. 2018;25(11):3308–15. 10.1245/s10434-018-6653-9 30083834 10.1245/s10434-018-6653-9

[39] Salem R, Gabr A, Riaz A, Mora R, Ali R, Abecassis M et al. Institutional decision to adopt Y90 as primary treatment for hepatocellular carcinoma informed by a 1,000-patient 15-year experience. Hepatology. 2018;68(4):1429–1440. 10.1002/hep.29691. Epub 2018 Jan 29. PMID: 29194711. 10.1002/hep.29691 29194711

[40] Tabone M, Calvo A, Russolillo N, Langella S, Carbonatto P, Lo Tesoriere R, Richetta E, Pellerito R, Ferrero A. Downstaging unresectable hepatocellular carcinoma by radioembolization using 90-yttrium resin microspheres: a single center experience. J Gastrointest Oncol. 2020;11(1):84–90. 10.21037/jgo.2019.06.01. PMID: 32175109; PMCID: PMC7052761. 32175109 10.21037/jgo.2019.06.01 PMC7052761

